# The impact of implant treatment on oral health related quality of life in a private dental practice: a prospective cohort study

**DOI:** 10.1186/1477-7525-11-197

**Published:** 2013-11-14

**Authors:** Mathieu Fillion, Dominique Aubazac, Marion Bessadet, Marlène Allègre, Emmanuel Nicolas

**Affiliations:** 1 Dom’Implant Formation, Clermont-Ferrand, France; 2 Clermont Université, Université d’Auvergne, EA4847, Centre de Recherche en Odontologie Clinique, BP 10448, Clermont-Ferrand F-63000, France; 3 CHU Clermont-Ferrand, Service d’Odontologie, Hôtel-Dieu, Clermont-Ferrand F-63000, France

**Keywords:** Dental implants, Oral Treatment, OHRQoL, Evaluation

## Abstract

**Background:**

Very few studies on the impact of implant therapy on Oral Health Related Quality of Life (OHRQoL) in partially edentulous patients have been published.

**Aim:**

This study aimed at analysing the improvement of OHRQoL of patients who underwent dental implant treatment using the “functional”, “psychosocial” and “pain and discomfort” categories of the Geriatric Oral Health Assessment Index (GOHAI).

**Methods:**

Within a prospective cohort of patients rehabilitated with Straumann dental implants, the OHRQoL of 176 patients (104 women and 72 men) was assessed using the GOHAI questionnaire, at two different times, before and after implant placement. The degree of oral treatment was categorised into three classes: “Single Tooth Implant” (n = 77), “Fixed Partial Denture” (n = 75), “Fixed or Retained Full Prostheses” (n = 24). The participants’ characteristics (gender, age, tobacco habits, periodontal treatment, time between both evaluations) were assessed.

**Results:**

Before treatment, the GOHAI score was lower for participants with fewer teeth (F = 19, P < 0.001). After treatment, no difference was observed between participants; significant improvements were observed in the GOHAI scores obtained (repeated measures, analysis, (F = 177, P < 0.001)) for each of the GOHAI fields studied (functional, psychosocial and pain & discomfort), regardless of the degree of treatment. The best improvement was observed in patients who needed complete treatment (P < 0.001). The presence of preliminary periodontal treatment, tobacco habits, age and gender of the participants did not have a significant impact on OHRQoL. Changing the time between the two evaluations (before and after treatment) had no impact on the changes in the GOHAI score.

**Conclusions:**

Implants enhanced the OHRQoL of participants that needed oral treatment.

## Background

Oral related quality of life could be defined as an individual’s perception of the impact of oral health on their quality of life (OHRQoL) [1]. OHRQoL is characterized by the individual’s perception of their position in life, in relation to their goals, expectations, standards, and concerns, and to the cultural conditions and the value system under which they live [2]. Some studies have demonstrated the significant relationship that exists between good oral quality of life and good general quality of life [3]. In this context, edentulism and conventional complete denture treatment have been shown to have a negative impact on OHRQoL [4]. The success or failure of oral treatment using conventional dentures depends on many factors, including the practitioner’s technical skills and unfavorable oral conditions [5]. The psychological aspect of treating edentulism is of great importance and the patient/practitioner relationship is essential [6]. Sometimes, despite the practitioner’s best efforts and full cooperation from the patient, it remains impossible to meet their expectations. For example, in a situation of bone crestal resorption the practitioner may propose dental implants [7].

Fifty years after the first oral treatment with titanium dental implants, the parameters conditioning osseointegration, such as oral hygiene, occlusal force and type of implant appear to be well-controlled. Rates of success and surgical procedures have been described extensively. Furthermore, many studies have explored the efficacy of implant treatment using objective parameters (retention, stability, chewing parameters): implants have been shown to improve denture stability and retention, consequently improving oral comfort and OHRQoL for patients [7,8]. However, so far, no clinical studies have measured the impact of implant fixed-prostheses on the OHRQoL of partially or completely edentulous participants. Practitioners can rely on a number of tools for evaluating OHRQoL, including the Oral Health Impact Profile (OHIP), the oral impacts on daily performances (OIDP), and the Geriatric Oral Health Assessment Index (GOHAI) [9]. These questionnaires explore the functional, social, and psychological impacts of oral disorders. The GOHAI is a 12-item assessment questionnaire originally developed by Atchison and Dolan in 1990 for studies on elderly populations [10], and it was later renamed the General Oral Health Assessment Index [11]. It has also been used in studies on younger adults [12]. The GOHAI has been validated in various languages [13-17], including French [18], unlike the OHIP and OIDP tools. Factors such as oral pain, denture dislodgements, and xerostomia can induce masticatory difficulties [19-21]. The GOHAI was shown to be sensitive to the provision of dental care [22], to be more appropriate when considering functional and psychosocial impacts, and to be more efficient at detecting changes in a participant than the OHIP [23,24] which is often used in OHRQoL evaluations [25]. The objective of this study was to assess whether implants and fixed or retained prostheses could improve the OHRQoL of partially or completely edentulous patients.

## Methods

### Evaluation process of OHRQol

For this study, OHRQol was assessed using the GOHAI questionnaire and data were collected during interviews. First, the validity of the GOHAI for this sample was controlled. The interviewer performed calibration beforehand and the reliability of this calibration was tested on five participants. GOHAI comprises 12 items grouped into three fields: (1) the functional field (eating, speaking, swallowing), (2) the psychosocial field (concerns, relational discomfort, appearance), (3) the pain or discomfort field (drugs, gingival sensitivity, discomfort when chewing certain foods). The method used in this study was the cumulative method (GOHAI-Add), which consists in adding the scores obtained for each of the 12 GOHAI questions. Each question is given a score between 1 and 5. The maximum score is 60 (20 = functional field; 25 = psychosocial field; 15 = pain or discomfort field). According to Atchison and Dolan (1990), a score ranging between 57 and 60 is considered as high and corresponds to a satisfactory OHRQol [10]. A score ranging between 51 and 56 is regarded as average, and a score of 50 or less is considered as a low score, reflecting a poor OHRQol.

### Description of participants

The participants included in the study had been referred to a private dental practice (France) over a one-year period (2010) for implant treatment. The sample size required was estimated from a previous study that measured the GOHAI-Add score in a group of edentulous participants rehabilitated with the implant procedure [26]. In this previous study, the GOHAI-Add scores increased from 43 ± 9 (before treatment) to 56 ± 3 (with implant). Therefore, for the present study, the calculations, completed with epiR package 0.9-30, were based on a difference of 13 points in GOHAI scores and a common standard deviation of 9. This indicated the need for at least 22 participants in each group studied (α = 5%, β = 10%). 176 participants were recruited (72 men and 104 women; mean age 52±9.9 years, mini = 18, maxi = 84). The participants were categorised according to the degree of oral treatment needed: “Single Tooth” (n = 77), “Fixed Partial Denture” (n = 75; 2 to 6 teeth replaced) and “Full Prostheses” (n = 24; 10 to 14 replaced teeth). The latter category comprised “full fixed Prostheses” (n = 11) and “Implant Retained Complete Over-denture” (n = 13). The characteristics of the participants were gathered and the distribution according to gender, age group, tobacco habits and potential preliminary periodontal treatment are presented in Table 1.

**Table 1:** **Distribution of participants**’ **characteristics according to each type of treatment**

		**Type of treatment**			**All categories**
		**Single tooth implant n (%)**	**Fixed partial denture n (%)**	**Full prostheses n (%)**	
Gender	Male	32 (41.6%)	31 (41.3%)	9 (37.5%)	72 (40.9%)
	Female	45 (58.4%)	44 (58.7%)	15 (62.5%)	104 (59.1%)
Age (years)	Less than 50	36 (46.8%)	15 (20%)	4 (16.7%)	55 (31.3%)
	50 to 60	25 (32.5%)	28 (37.3%)	4 (16.7%)	57 (32.4%)
	More than 60	16 (20.7%)	32 (42.7%)	16 (66.6%)	64 (36.3%)
Periodontal treatment	Yes	15 (19.5%)	12 (16%)	3 (12.5%)	30 (17%)
	No	62 (80.5%)	63 (84%)	21 (87.5%)	146 (83%)
Tobacco habits	Yes	11 (14.3%)	16 (21.3%)	4 (16.7%)	31 (17.6%)
	No	66 (85.7%)	59 (78.7%)	20 (83.3%)	145 (82.4%)
Time between two evaluations (months)	Less than 6	19 (24.7%)	26 (34.7%)	9 (37.5%)	54 (30.7%)
	6 to 9	31 (40.2%)	22 (29.3%)	9 (37.5%)	62 (35.2%)
	More to 9	27 (35.1%)	27 (36%)	6 (25%)	60 (34.1%)

Fixed partial denture replaced 2 to 6 teeth and full prostheses 10 to 14 teeth.

The aim, benefits, and risks of the experiment were explained to the participants and each of them signed a consent form. These patients were assessed for their OHRQoL by answering a GOHAI questionnaire [10] before implant placement and between 3 months and 15 months after treatment (average; 7.3±3.1 months). Three homogenous groups were then formed according to the time between two evaluations: less than 6 months (n = 54), 6 to 9 months (n = 62), and more to 9 months (n = 60). Gender, age, tobacco habits and potential preliminary periodontal treatment were also determined at the beginning of the study.

Participants that were excluded from the study: 1) presented cognitive deficiency, 2) were dependent or living in institutions, or 3) had stopped treatment prematurely.

### Data acquisition and analysis

Statistical analysis was performed using SPSS 20 software (SPSS Inc., Chicago, IL). Before analysis, the validity of the study sample was verified. The purpose of the analysis was to verify that oral treatment would improve OHRQoL and level the difference between the degrees of tooth loss. Data from the GOHAI questionnaire before and after implant treatment were analyzed using a General Linear Model procedure (GLM) (**variable**: GOHAI parameters; **fixed variable**: type of oral treatment). A Student Newman-Keuls Post Hoc test (SNK) was applied to discriminate the impact of the type of treatment (α = 0.05). The same method was used to measure possible impacts of age, gender, primary periodontal treatment and tobacco habits on GOHAI scores before and after treatment. The impact of the type of treatment and different follow-up times on GOHAI scores was tested by repeated-measure analysis (**variable**: GOHAI parameters before and after treatment; **fixed variable**: type of oral treatment; follow up time) followed by the SNK test. Possible impacts of age, gender, periodontal treatment and tobacco habits on GOHAI scores were tested similarly.

## Results

### Validity of the instrument

The validity of the GOHAI in this sample was assessed for (i) concurrent validity: before treatment, GOHAI scores were significantly lower in patients with poor oral health perception (44.5 ± 9.1) and higher in participants with good oral health perception (49.7 ± 6.7) (t-test, P < 0.01); (ii) reliability, assessed using Cronbach’s alpha. High reliability was found between the different items of the GOHAI questionnaire before treatment (Cronbach’s alpha = 0.80) and after treatment (Cronbach’s alpha = 0.76); (iii) discriminant validity: the GOHAI questionnaire was able to discriminate between participants with different oral health status before treatment. GOHAI mean scores were significantly lower in edentulous participants (40.4 ± 11.6), moderate in participants presenting partial edentulism (48.1 ± 11.6), and higher in participants with single edentulism (50.0 ±7.09) (Anova, P < 0.01).

### GOHAI scores

For each evaluation time (before and after treatment), the mean values (±SD) of GOHAI-Add and each GOHAI field (Functional, Pain or Discomfort, and Psychosocial domains) are presented in Table 2 according to gender, age, tobacco habits and preliminary periodontal treatment. The characteristics of this population did not have any statistically significant impact on GOHAI-Add scores before or after treatment. These parameters were also not associated with changes in the OHRQoL of the participants.

**Table 2:** **Means values** (±**SD**) **of GOHAI**-**Add**, **for each GOHAI fields**, **before and after treatment and according to the gender**, **age**, **tobacco habits and primary periodontal treatment**

		**GOHAI add**		**Functional field**		**Pain and discomfort field**		**Psychosocial field**	
		**Before**	**After**	**Before**	**After**	**Before**	**After**	**Before**	**After**
Gender	Female	47.6	54.5	16.7	18.8	11.4	13.3	19.5	22.5
		(8.4)	(4.3)	(3.3)	(1.6)	(2.1)	(1.7)	(4.2)	(2.1)
	Male	49.2	55.0	16.9	18.8	11.9	13.6	20.4	22.6
		(7.4)	(4.5)	(2.9)	(1.5)	(2.0)	(1.5)	(3.7)	(2.3)
Age	Less than 50 years	49.1	55.2	17.1	19.1	11.8	13.5	20.2	22.7
		(8.3)	(3.8)	(3.0)	(1.3)	(2.0)	(1.7)	(4.1)	(1.9)
	50 to 60 years	49.0	54.0	17.2	18.7	11.6	13.2	20.2	22.1
		(6.7)	(4.6)	(2.6)	(1.5)	(2.1)	(1.6)	(3.4)	(2.3)
	More than 60 years	46.8	55.0	16.1	18.6	11.5	13.6	19.3	22.8
		(8.9)	(4.5)	(3.6)	(1.7)	(2.1)	(1.5)	(4.5)	(2.1)
Tobacco habits	No	48.5	55.0	16.8	18.8	11.7	13.5	20.0	22.6
		(8.3)	(4.3)	(3.2)	(1.6)	(2.1)	(1.6)	(4.1)	(2.1)
	Yes	47.3	53.6	16.8	18.7	11.5	12.8	18.9	22.1
		(6.9)	(4.3)	(2.9)	(1.3)	(1.7)	(1.5)	(3.9)	(2.4)
Periodontal treatment	No	48.6	54.7	16.8	18.8	11.7	13.4	20.1	22.5
		(8.0)	(4.3)	(3.2)	(1.5)	(2.1)	(1.5)	(4.0)	(2.1)
	Yes	46.7	54.8	16.4	18.8	11.5	13.5	18.8	22.6
		(8.4)	(4.5)	(3.2)	(1.5)	(2.0)	(1.8)	(4.2)	(2.2)

Before treatment, the GOHAI mean score for all the participants was 48, reflecting a poor OHRQoL (<50) [10]. For the patients scheduled for single unit treatment, the GOHAI-Add was 51, showing an average OHRQoL [10]. The mean scores according to the type of treatment (single, partial fixed denture or full prostheses) and the duration between two evaluations (less than 6 months, 6 to 9 months or more) are given in Table 3.

**Table 3:** **Mean values** (±**SD**) **of GOHAI**-**add**, **for the functional**, **pain and discomfort and psychosocial fields**, **before and after treatment in each group of participants**

		**GOHAI-add**		**Functional field**		**Pain and discomfort field**		**Psychosocial field**	
**Type of treatment**	**Follow-up time (months)**	**Before**	**After**	**Before**	**After**	**Before**	**After**	**Before**	**After**
Single tooth implant	less than 6	50.1	53.5	17.6	18.7	11.4	12.8	21.1	22.2
		(7.3)	(4.9)	(2.9)	(1.6)	(2.0)	(1.8)	(3.1)	(2.2)
	6 to 9	51.8	55.5	17.7	19.2	12.5	13.7	21.5	22.8
		(5.1)	(3.8)	(2.0)	(1.3)	(1.7)	(1.7)	(2.8)	(2.1)
	more to 9	50.4	55.2	17.8	19.3	11.9	13.4	20.8	22.6
		(8.6)	(4.6)	(2.9)	(1.2)	(2.3)	(1.7)	(4.2)	(2.4)
	Total	50.0	54.9	17.7	19.1	12.0	13.4	21.2	22.6
		(7.0)	(4.4)	(2.5)	(1.4)	(2.0)	(1.7)	(3.4)	(2.2)
Fixed partial denture	less than 6	48.1	54.6	17.0	18.8	11.4	13.4	19.7	22.4
		(6.8)	(4.2)	(2.9)	(1.5)	(2.3)	(1.7)	(3.2)	(2.0)
	6 to 9	50.1	54.8	17.9	18.9	11.5	13.1	20.7	22.8
		(5.5)	(4.8)	(2.0)	(1.5)	(1.8)	(1.6)	(3.0)	(2.5)
	more to 9	46.3	54.4	15.9	18.8	11.5	13.4	18.9	22.5
		(4.8)	(4.6)	(1.9)	(1.8)	(1.6)	(1.4)	(3.0)	(2.1)
	Total	48.1	54.6	16.9	18.7	11.4	13.3	19.7	22.5
		5.9	(4.5)	(2.4)	(1.6)	(1.9)	(1.5)	(3.1)	(2.2)
Full prosthese	less than 6	36.0	53.1	11.9	17.6	10.8	13.8	13.3	21.8
		(10.4)	(3.7)	(4.4)	(1.1)	(2.3)	(1.1)	(4.9)	(2.0)
	6 to 9	43.0	54.7	14.2	18.2	10.8	14.1	18.0	22.3
		(12.9)	(4.8)	(4.7)	(2.0)	(2.6)	(1.2)	(6.7)	(2.4)
	more to 9	43.2	56.3	14.7	19.5	11.5	13.7	17.0	23.2
		(11.2)	(2.2)	(4.9)	(0.8)	(2.8)	(1.8)	(5.1)	(1.3)
	Total	40.4	54.5	13.5	18.37	11.0	13.9	16.0	22.3
		(11.6)	(3.9)	(4.6)	(1.6)	(2.5)	(1.3)	(5.8)	(2.0)
Total	less than 6	46.8	54.0	16.4	18.6	11.3	13.2	19.1	22.2
		(9.0)	(4.4)	(3.7)	(1.6)	(2.2)	(1.7)	(4.4)	(2.1)
	6 to 9	49.9	55.1	17.3	18.9	11.9	13.5	20.7	22.7
		(7.3)	(4.3)	(2.8)	(1.5)	(2.0)	(1.6)	(3.7)	(2.2)
	more to 9	47.9	55.0	16.6	18.9	11.7	13.4	19.6	22.6
		(7.7)	(4.4)	(2.9)	(1.5)	(2.1)	(1.5)	(3.9)	(2.2)
	Total	48.3	54.7	16.8	18.8	11.6	13.4	19.8	22.5
		(8.1)	(4.3)	(3.2)	(1.5)	(2.1)	(1.6)	(4.0)	(2.1)

Fixed partial denture replaced 2 to 6 teeth and full prothese 10 to 14 teeth.

Before treatment, the mean GOHAI scores were lower for participants with fewer teeth (F = 19, P < 0.001): the SNK test showed that the mean GOHAI score of participants scheduled for single and partial treatment was better than that of patients in need of complete treatment (Table 3). Similar results were found for the functional (F = 20, P < 0.001) and psychosocial (F = 18, P < 0.001) fields, but not for the “pain or discomfort” fields. After treatment, the GOHAI mean score of all 176 participants reached 54, which corresponds to an average OHRQoL. The variability of the score was reduced: no difference between the types of treatment was observed, regardless of the GOHAI field. Whatever the follow-up time, significant improvements were observed in the GOHAI-Add scores and in the scores for each GOHAI field (Table 4). The change in the GOHAI score was greater for the patients with the fewest teeth, and the best improvement was noted for complete treatment (P < 0.001; Effect size = 0.36) compared with single tooth implants (P < 0.001; effect size = 0.13) and fixed partial dentures (P < 0.001; effect size = 0.27).

**Table 4:** **Repeated measure analysis on GOHAI**-**Add values** (**before and after rehabilitation**) **and for each GOHAI fields**

	**GOHAI-add**				**Functional field**				**Pain and discomfort field**				**Psychosocial field**			
	***ddl***	***F***	***P***	***Effect size***	***ddl***	***F***	***P***	***Effect size***	***ddl***	***F***	***P***	***Effect size***	***ddl***	***F***	***P***	***Effect size***
**Impact of treatment**	**1**	**177**	<**0.001**	**0.51**	**1**	**137**	<**0.001**	**0.45**		**124**	<**0.001**	**0.42**	**1**	**133**	<**0.001**	**0.44**
Impact of treatment* Type (λ)	2	18	<0.001	0.18	2	17	<0.001	0.18	2	5	<0.01	0.54	2	18	<0.001	0.18
Impact of treatment* Follow-up time (λ)	2	1	ns	0.02	2	1	ns	0.01	2	0	ns	0.02	2	2	ns	0.03

Statistical analyses were used to test the impact of the type of treatments (single tooth implants, fixed partial denture, full prostheses) and the different follow-up times on GOHAI values.

ns non significant.

λ Interactions between two factors.

## Discussion

The main objective of this study was to highlight the impact of dental implant treatment on the OHRQoL of a group of patients. The data collected showed that OHRQoL was improved after implant treatment, regardless of the GOHAI fields measured.

The evaluation tool used most commonly in many studies conducted on the OHRQoL of patients is the OHIP (Oral Health Impact Profile) [1,24,25]. However, the OHIP has not been validated in French and therefore could not be used for this study. For similar reasons, the Visual Analogue Scales (VAS) used in many studies to assess the OHRQoL of patients could not be used in the present study. However, a validated French version of the GOHAI was available [18]. The GOHAI simply consists in a series of clear and concise questions grouped in twelve items which allow accurate analysis of the functional and psychosocial domains and of discomfort and pain. This questionnaire is reproducible, easy to use and has already been applied previously in the evaluation of the impact of oral treatment [26,27]. After taking into account all these considerations, the GOHAI was used for this study. The methodology used in the present study is similar to that of previous studies [26-28], though it presents some biases and weaknesses. First, the lack of investigator calibration could be a limitation. Furthermore, the patients included in this study originated from a single dental practice and it is not possible to determine whether the results are practitioner-dependent. Furthermore, the long-term impact of implant treatment on the OHRQoL could not be measured due to the fact that patient follow-up did not exceed 2 years. Similarly, only the type of edentulousness was considered but not the different surgical procedures that were performed (Immediate loading, post-extractional, etc.). Additional studies should be conducted to improve these points.

Before treatment, the results also displayed great variability in the GOHAI scores according to the type of edentulousness: the greater the need for treatment, the lower the GOHAI scores. This variability was present within every type of edentulousness, in particular with participants scheduled for complete treatment. Therefore, the OHRQoL of the patients was very variable. These results confirm a previously published study [4]. Three months after implant treatment, the GOHAI-Add score for all the patients increased and displayed less variation, regardless of the type of treatment. The GOHAI mean score reached 54 while the change in the GOHAI score was greatest for the patients with the fewest teeth.

Implant treatment led to an improvement of OHRQoL in the three fields of the GOHAI (functional, psychosocial, discomfort and pain). Before treatment, the functional GOHAI score was lower for those with the fewest teeth but, afterwards, scores varied less and were no longer significantly different. Indeed, participants presented the same masticatory and phonetic abilities, regardless of the type of treatment performed. As with previous studies, these results confirmed the physiological and functional benefits of implant treatment [26,29,30]. With respect to the psychosocial field, the GOHAI scores before treatment were significantly lower for those with fewer teeth (P < 0.001). After treatment, these scores were no longer statistically different according to the type of prosthetic treatment (P < 0,001). A low GOHAI score in the psychosocial field reflects a difficulty for maintaining regular social relationships, embarrassment at eating in front of other people, concerns over dental, and/or gingival status, or over dentures. In particular, the treatment greatly improves the everyday life of complete denture wearers [27,31,32]. Initially, exploration of the “discomfort and pain” field did not reveal any significant difference between the different types of edentulousness. A significant improvement of these scores was observed after treatment. This field studied comfort during meals, consumption of analgesics and sensitivity of teeth or gums to warmth, and cold and sweet food. The scores before treatment were low and confirmed the need for care for all of the participants included in the study.

This study emphasized the fact that the OHRQoL of people who underwent single implant treatment was improved. These results agree with those of a previous study [33] in which 90% of the patients evaluated with a VAS were satisfied by implant treatment when considering the aesthetic and functional points of view. With regards to partial treatment by implants, a significant improvement of OHRQoL was observed, thus corroborating results obtained in another study that measured OHRQoL using the OHIP. Similarly, the OHRQoL of the partially dentate participants was lower than that of fully dentate participants [34]. The present work also showed a significant improvement of OHRQoL in retained or fixed complete denture wearers, as previously reported in another study, which used the OHIP and OIDP as evaluation tools [31]. In addition, two studies using the OHIP did not demonstrate any difference in OHRQoL between conventional and implant-retained complete denture wearers [35,36]. On the other hand, before treatment, participants that accepted implant treatment presented an initially poorer OHRQoL than that of conventional full removable denture wearers. Subsequently, the improvement of their OHRQoL was also greater. Two metanalyses calculated comparable results [30,37]. It is possible that the sole fact of receiving an implant treatment has a positive and subjective impact on individuals. For a number of patients, this “modern” technique is the best way to improve their oral state and thus to improve their OHRQoL. Moreover, it has also been shown that treatment with conventional or implant-retained dentures has an impact on social and sexual activities. Two months after treatment, participants wearing full dental implants showed higher OHIP scores, especially in the following activities; eating, speaking, kissing, and yawning [38].

The GOHAI scores after treatment were similar for individuals who underwent fixed or removable complete treatment. This was already demonstrated in previous studies using OHIP as a tool [32,39]. Finally, all the patients were satisfied about their chewing ability and the aesthetics. Maintaining oral hygiene was easier for the wearers of removable prostheses on implants. However, regarding the psychosocial aspect, participants wearing a fixed prosthesis were generally more satisfied than others, as previously described by Brennan and co-workers (2010) [32]. More often, the improvement of OHRQoL would be better with a fixed prosthesis [32]. However, a study using VAS showed improved oral quality of life for removable treatments on implants [29].

## Conclusions

OHRQoL was globally improved after oral treatment by implants. However, a study with a longer follow-up period would be necessary to validate the long-term benefits of oral implantology with regards to OHRQoL. Similarly, studying the possible effects of various implant treatment techniques would be of great interest.

## Competing interest

The authors declare that they have no competing interests.

## Authors’ contributions

The study was conceived and designed by: MF, DA, EN. Data was collected by: MF, DA, MA. Data were analyzed by: EN, MB. Materials and analysis tools were contributed by: MF, DA. Authors: MF, EN. All authors read and approved the final manuscript.
